# Occurrence of spintronics behaviour (half-metallicity, spin gapless semiconductor and bipolar magnetic semiconductor) depending on the location of oxygen vacancies in BiFe_0.83_Ni_0.17_O_3_

**DOI:** 10.1098/rsos.170273

**Published:** 2017-06-14

**Authors:** P. Iyyappa Rajan, S. Mahalakshmi, Sharat Chandra

**Affiliations:** 1 Chemistry Division, School of Advanced Sciences, Vellore Institute of Technology (VIT) University, Chennai Campus, Vandalur–Kelambakkam Road, Chennai 600127, India; 2 Materials Science Group, Indira Gandhi Centre for Atomic Research (IGCAR), Kalpakkam, Tamil Nadu 603102, India

**Keywords:** oxygen vacancies, spintronics, ferrimagnetic

## Abstract

The current communication signifies the effect of oxygen vacancies (OVs) both qualitatively and quantitatively in multiferroic BiFe_0.83_Ni_0.17_O_3_ by an in-depth atomic-level investigation of its electronic structure and magnetization properties, and these materials have a variety of applications in spintronics, optoelectronics, sensors and solar energy devices. Depending on the precise location of OVs, all the three types of spintronic material namely half-metallic, spin gapless semiconductor and bipolar magnetic conductor have been established in a single material for the first time and both super-exchange and double-exchange interactions are possible in accordance with the precise location of OVs. We have also calculated the vacancy formation energies to predict their thermodynamic stabilities. These results can highlight the impact and importance of OVs that can alter the multiferroic properties of materials.

## 1. Introduction

Recently, multiferroic materials have been perceived as an ideal candidate for novel applications that include but are not limited to spintronics, magnetic field sensors and multiple state memory elements [1]. Researchers are making extensive efforts in fabricating a robust, high performance and relatively less energy expensive memory storage device as a result of huge increasing technological demand over the past decade. Consequently, multiferroic materials surge in importance for the current technological evolution as it shows significant coupling of ferroelectricity and ferromagnetism specifically at room temperature. Bismuth ferrite (BiFeO_3_-BFO), a class of single-phase multiferroic materials is one of the most promising material for next-generation technological devices owing to its very high both antiferromagnetic Neel temperature of approximately 640 K and ferroelectric Curie temperature of approximately 1100 K [1]. However, ferroelectric polarization measurements of BFO samples show high electrical leakage currents thereby limiting its applications in memory storage devices [2]. It has been well established that by means of site-engineering approach (doping at Bi and Fe sites) the leakage current can be significantly controlled [3,4] and in particular, doping of aliovalent ions at Fe site greatly influences the electronic structure, releases net magnetization by destroying the cycloid-type magnetic structure and enhances the optical properties of BFO [5]. One of the most successful aliovalent ions doped in BFO is examined to be Ni^2+^ and experimental reports of Ni-doped BFO shows enhanced multiferroic properties [6,7] and also predict that the introduction of Ni^2+^ is expected to create more oxygen vacancies (OVs) and prevent the formation of Fe^2+^ ions. Our previous density functional theory (DFT) calculations on stoichiometric BiFe_0.83_Ni_0.17_O_3_ (approx. 16.67 at% of Ni) with zero OVs displayed half-metallic behaviour which holds applications in spintronics [8]. Nevertheless, it is very difficult to determine half-metallicity by experimental measurements owing to various reasons which includes uncertainty in measurements, incomplete spin polarization [9], and existence of structural disorders [10,11]. In particular, OVs do occur in the synthesis of BFO samples and both qualitative and quantitative presence of OVs play a vital role in modulating the electronic, magnetic and optical properties of Ni-doped BFO. Nevertheless, the accurate and in-depth role of OVs is still puzzling at an atomic level and an understanding of the modulated behaviour of Ni-doped BFO is required, therefore in this short communication, we have tried to address the influence of OVs both nearer and farther to Ni ion in BiFe_0.83_Ni_0.17_O_3_. By employing first principles DFT calculations, we have made an effort in exploring the behaviour of BiFe_0.83_Ni_0.17_O_3_ by varying the OVs concentration both qualitatively and quantitatively. The details of the calculations performed are mentioned below in the computational methodology section.

## 2. Computational methodology

**Figure 1.**
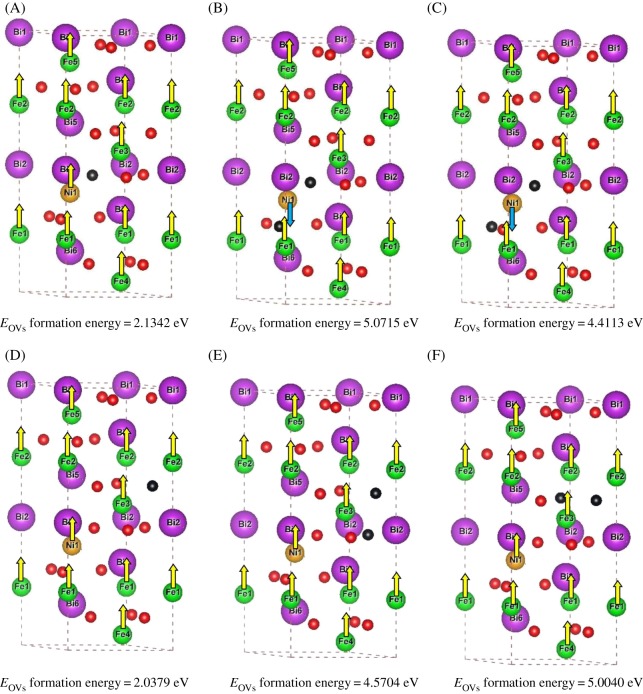
Modelled hexagonal cells of BiFe_0.83_Ni_0.17_O_3_ with six various OVs configurations given with their calculated vacancy formation energies and ground state magnetic configurations designated as: (i) 1 OV nearer to Ni (A), (ii) 1 OV nearer to Ni and second OV nearer to first OV (B), (iii) 1 OV nearer to Ni and second OV farther to first OV (C), (iv) 1 OV farther to Ni (D), (v) 1 OV farther to Ni and second OV nearer to first OV (E), and (vi) 1 OV farther to Ni and second OV farther to first OV (F) (violet spheres are Bi, green spheres are Fe, red spheres are O and black spheres are OVs).

In our previous calculations [8], we have modelled a hexagonal cell having molecular formula BiFe_0.83_Ni_0.17_O_3_ containing 30 atoms (6 Bi atoms, 5 Fe atoms, 1 Ni atom and 18 O atoms) which include six formula units of BFO. In the above hexagonal cell, we have created OVs concentration in the range of 5.56 (1 OV) and 11.11 at% (2 OVs) by removing one and two O atoms both nearer and farther to the nickel atom. In this manner, we have modelled six various configurations and designated as: (i) 1 OV nearer to Ni (A), (ii) 1 OV nearer to Ni and second OV nearer to first OV (B), (iii) 1 OV nearer to Ni and second OV farther to first OV (C), (iv) 1 OV farther to Ni (D), (v) 1 OV farther to Ni and second OV nearer to first OV (E), and (vi) 1 OV farther to Ni and second OV farther to first OV (F) as shown in figure 1. The input file coordinates of the perfect hexagonal cell BiFe_0.83_Ni_0.17_O_3_ containing 30 atoms was deposited in Dryad and the configuration (A) can be generated by removing 1 O atom in the nearest neighbourhood of an Ni atom with coordinates (0.43653(*x*), 0.42024(*y*), 0.45788(*z*)) in this file. Similarly, (B) can be generated by removing 2 O atoms in the same neighbourhood with a distance of 2.8016 Å between them, with the coordinates (0.43653(*x*), 0.42024(*y*), 0.45788(*z*)) and (0.10427(*x*), 0.34381(*y*), 0.29099(*z*)). Configuration (C) can be generated by removing 2 O atoms at a distance of 3.9804 Å between them and with the coordinates (0.43653(*x*), 0.42024(*y*), 0.45788(*z*)) and (0.23954(*x*), 0.89573(*y*), 0.29099(*z*)). Similarly, the configuration (D) has been generated by removing 1 O atom with coordinates (0.90944(*x*), 0.23022(*y*), 0.62273(*z*)) which is not near an Ni atom, configuration (E) is generated by removing 2 O atoms at a distance 2.7846 Å with the coordinates (0.90944(*x*), 0.23022(*y*), 0.62273(*z*)) and (0.98371(*x*), 0.56347(*y*), 0.45788(*z*)) and the configuration (F) can be generated by removing 2 O atoms at a distance of 2.9111 Å with coordinates (0.90944(*x*), 0.23022(*y*), 0.62273(*z*)) and (0.76978(*x*), 0.67922(*y*), 0.62273(*z*)). DFT calculations were performed for all the modelled configurations by using Vienna Ab-initio Simulation package [12,13]. The projector augmented wave method [13,14] was adopted in our calculations by considering the valence electrons for Bi (6s^2^6p^3^), Fe (3d^6^4s^2^), Ni (3d^8^4s^2^) and O (2s^2^2p^4^). The generalized gradient approximation (GGA) as a revised version of Perdew, Burke and Ernzerhof [15] was employed to treat the exchange and correlation effects of electrons. A plane wave kinetic energy cutoff for the plane wave basis set of 500.00 eV was used throughout the calculations and denser Γ-centred k-point mesh density of 4 × 4 × 1 was generated for the integration of Brillouin zone. Relaxation of the ionic positions are stopped until the Hellmann–Feynman forces are less than 10^−2^ eV/Å and the SCF iterations are completed with the total energy convergence of 10^−6^ eV. The relaxation of all the configurations was carried out by means of conjugate gradient method [16] with Gaussian broadening of 0.1 eV. The strong correlations effects have been treated by performing GGA + U calculations and in our calculations, we have followed the approach of Dudarev *et al*. [17] where an effective Hubbard parameter U_eff_ = U-J enters the Hamiltonian, by considering J as the exchange interaction parameter. The effective Hubbard parameter U_eff_ for Fe is fixed to 4.00 eV and for Ni is fixed to 5.00 eV, in accordance with our previous calculations [8].

## 3. Results and discussion

**Figure 2.**
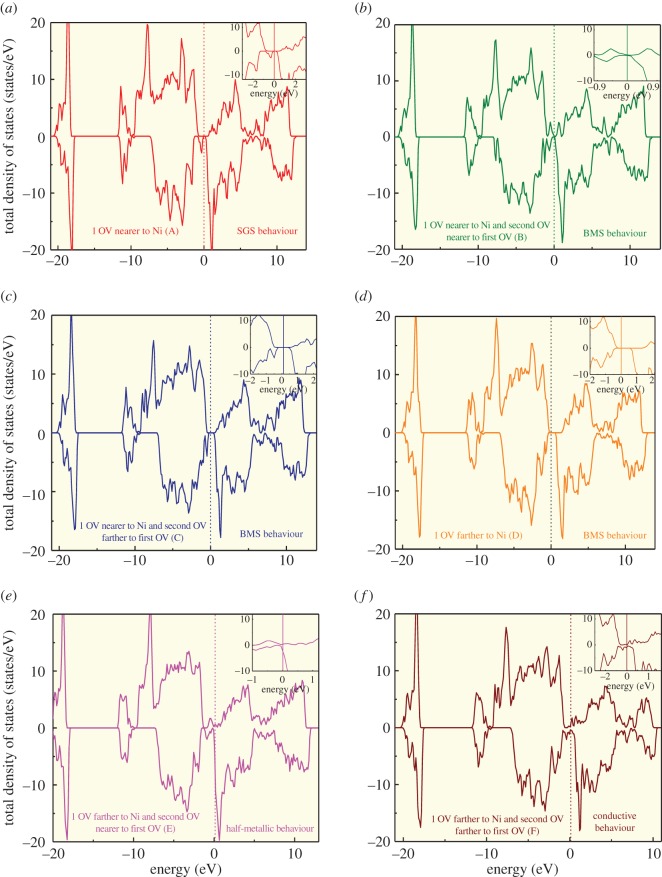
Total density of states calculated after the relaxation of six modelled hexagonal cells of BiFe_0.83_Ni_0.17_O_3_ with six various OVs configurations: (*a*) 1 OV nearer to Ni (A), (*b*) 1 OV nearer to Ni and second OV nearer to first OV (B), (*c*) 1 OV nearer to Ni and second OV farther to first OV (C), (*d*) 1 OV farther to Ni (D), (*e*) 1 OV farther to Ni and second OV nearer to first OV (E) and (*f*) 1 OV farther to Ni and second OV farther to first OV (F).

The structural modifications which include bond angles, bond lengths, lattice parameters and cell volume of all the configurations with OVs will be discussed elaborately in our next article. In the current communication, we are reporting our calculated results only about the thermodynamic stability of the modelled configurations with OVs and their electronic and magnetic properties. The thermodynamic stability of the modelled configurations was determined by calculating their OVs formation energies, respectively, and is given in figure 1. Formation energy is lower for the (D) configuration in which the OV is farther to nickel atom (1 OV) and indicates the greater stability of D compared with all other configurations. Meanwhile, the formation energies are lower for BiFe_0.83_Ni_0.17_O_3_ with single OV with 5.56 at% (A and D) rather than with two OVs with 11.11 at% (B, C, E and F) and shows that increase in OVs destabilizes the system. The above results were also pointed out in the photoluminescence measurements of Ni-doped BiFeO_3_ [18] in which a stable structure requires only limited concentration of OVs. We have found that the magnetization values of Fe and Ni ions are influenced by the presence and precise location of OVs. The magnetic moment values of Fe and Ni atoms are listed in table 1, respectively, and the existence of both Fe^2+^ ions with approximately 3.7–3.9 µB and Fe^3+^ ions with more than 4.0 µB was observed in all the configurations except (A) in which only Fe^3+^ ions exist (when 1 OV is nearer to Ni). When the higher concentration of OVs (11.11 at%) are nearer to Ni atom as in (B) and (C) configurations, ferrimagnetism is displayed and the Fe and Ni ions are oppositely aligned. Super-exchange interactions are possible via Fe^2+^–O^2−^–Ni^2+^ hybridization. When the concentration of OVs are located farther to the Ni atom (both 5.56 and 11.11 at%), Fe and Ni atoms shows ferrimagnetic moments with parallel alignment of Fe and Ni atoms and displays double-exchange interactions via Fe^3+^–O^2−^–Ni^2+^ interactions. The calculated net magnetic moment for the stoichiometric BiFe_0.83_Ni_0.17_O_3_ from our previous calculation was about 27.08 µB. However, when 1 OV is nearer to Ni (A), the net magnetic moment is slightly enhanced to 27.315 µB and when 1 OV is farther to Ni (D) there is no significant change in the magnetic moment. But when the concentration of OVs are increased, the net magnetic moments decreases as shown in table 1. Also it is clearly evident that the oppositely aligned super-exchange interactions in (B) and (C) configurations will decrease the net magnetic moment to a greater extent than the parallel alignment of Fe and Ni atoms through double-exchange interactions in (E) and (F) configurations. The electronic density of states was calculated for all the configurations as shown in figure 2, and it is confirmed from our plots that the both qualitative and quantitative presence of OVs have a strong impact on the Fermi level of electronic structure of BiFe_0.83_Ni_0.17_O_3_. All the types of spintronic materials behaviour [19] namely, half-metal (HM), spin gapless semiconductor (SGS) and bipolar magnetic semiconductor (BMS) are exhibited depending on the OVs concentration and its precise location and for the very first time, to our knowledge, we have predicted that the establishment of any kind of spintronics behaviour can be created by the precise control of OVs. In our previously published results [8], we have established half-metallicity in stoichiometric BiFe_0.83_Ni_0.17_O_3_. In the current work, when 1 OV is nearer to Ni (A), the electronic density of states shows spin gapless semiconducting behaviour when the up spin channel is semiconducting and the down spin channel is gapless. The band gap of the up spin channel is 1.5 eV and an almost zero band gap is shown in the down spin channel for configuration (A) with OV concentration of 5.56 at% in figure 2*a*. The configurations (B) and (C) with the increased OVs concentration (11.11 at%) nearer to Ni shows bipolar magnetic semiconducting behaviour with two different band gaps in the up and down spin channels as shown in figure 2*b,c*. Interestingly, when 1 OV is farther to the Ni (D), once again BMS behaviour arises with different band gaps in up (1.9 eV) and down spin (1.0 eV) channels which can be noticed in figure 2*d*. However, when the OVs concentration is 11.11 at% (2 OVs) farther to the Ni, configuration (E) shows half-metallic behaviour and configuration (F) shows conductivity as shown in figure 2*e,f*. These results clearly predicts that when the OVs are created and increased nearer to the Ni ion in BiFe_0.83_Ni_0.17_O_3_ (A, B and C) there is a transition from SGS to BMS with increasing band gaps in both the spin channels. At the same time, when the OVs are created and increased farther to the Ni ion in BiFe_0.83_Ni_0.17_O_3_ (D, E and F) there is a transition from BMS to conductivity with vanishing band gaps in both the spin channels. Previously, transport properties measurements of Ni-doped BFO films with significant OVs have shown enhanced conductivity in which the OVs can form deep-trap energy levels in which the electrons tend to be free charge carriers in the Fermi level [5]. Ultimately, Ni-doped BFO samples undergo charge compensation by formation of OVs and hence increase in the concentration of OVs results in conductivity. The respective band gaps at the Fermi level wherever applicable in all the configurations are given in table 2 and hence we have proved from our calculated results that OVs can alter the electronic Fermi level of density of states and this controlled tuning of OVs can induce the creation of all the types of spintronics behaviour and also alter the magnetic arrangement in multiferroic materials.

**Table 1.** Magnetic moments (µB) of Fe and Ni atoms and the total magnetic moments (µB) of modelled hexagonal cells calculated after the relaxation of modelled hexagonal cells of BiFe_0.83_Ni_0.17_O_3_ with six various OVs configurations.

	magnetic moments (µB) of Fe and Ni atoms						
configuration	Fe(1)	Fe(2)	Fe(3)	Fe(4)	Fe(5)	Ni(1)	total magnetic moments (µB) of modelled hexagonal cells (includes also residual magnetic moments of Bi and O atoms)
1 OV nearer to Ni (A)	4.594	4.589	4.254	4.618	4.615	1.883	27.315
1 OV nearer to Ni and second OV nearer to first OV (B)	3.856	4.599	4.352	4.589	4.612	−1.655	22.700
1 OV nearer to Ni and second OV farther to first OV (C)	3.864	4.587	3.886	4.597	4.609	−1.030	22.661
1 OV farther to Ni (D)	4.619	4.523	3.854	4.611	4.617	2.009	27.094
1 OV farther to Ni and second OV nearer to first OV (E)	4.504	4.468	3.822	4.523	4.526	1.726	26.369
1 OV farther to Ni and second OV farther to first OV (F)	4.586	3.792	3.721	4.588	4.544	1.949	25.201

**Table 2.** Band gap values of up and down spin channels of BiFe_0.83_Ni_0.17_O_3_ with six various OVs configurations and their electronic behaviour at the Fermi level from the total density of states.

configuration	electronic behaviour at the Fermi level in total density of states	up and down spin channel band gaps (eV)
1 OV nearer to Ni (A)	SGS	1.5, 0.2
1 OV nearer to Ni and second OV nearer to first OV (B)	BMS	0.6, 0.4
1 OV nearer to Ni and second OV farther to first OV (C)	BMS	1.6, 0.8
1 OV farther to Ni (D)	BMS	1.9, 1.0
1 OV farther to Ni and second OV nearer to first OV (E)	HM	0.4, 0.0
1 OV farther to Ni and second OV farther to first OV (F)	conductivity	0.0, 0.0

## 4. Conclusion

In summary, we present the impact of OVs both nearer and farther to the Ni ion in BiFe_0.83_Ni_0.17_O_3_ and its existence will modulate both the electronic structure and magnetization properties of BiFe_0.83_Ni_0.17_O_3_ significantly according to their precise locations, and these results can boost up the further investigation in experiments. The existence of half-metallicity, SGS and BMS in BiFe_0.83_Ni_0.17_O_3_ depends on the exact location of OVs. We have found the formation energy to be lowest for the configuration in which OV is farther away from the nickel atom indicating greater stability of this configuration compared to others. Further work is heading towards the impact of OVs on its charge density patterns with bonding, optical and electrical properties.

## Data accessibility

The manuscript data were deposited at the Dryad Digital Repository: (http://dx.doi.org/10.5061/dryad.1qs7p) [20].
